# Involvement of FATP2-mediated tubular lipid metabolic reprogramming in renal fibrogenesis

**DOI:** 10.1038/s41419-020-03199-x

**Published:** 2020-11-20

**Authors:** Yuting Chen, Qi Yan, Mengyue Lv, Kaixin Song, Yue Dai, Yi Huang, Le Zhang, Cuntai Zhang, Hongyu Gao

**Affiliations:** Department of Geriatrics, Tongji Hospital, Tongji Medical College, Huazhong University of Science and Technology, Wuhan, China

**Keywords:** Mechanisms of disease, Molecular biology

## Abstract

Following a chronic insult, renal tubular epithelial cells (TECs) contribute to the development of kidney fibrosis through dysregulated lipid metabolism that lead to lipid accumulation and lipotoxicity. Intracellular lipid metabolism is tightly controlled by fatty acids (FAs) uptake, oxidation, lipogenesis, and lipolysis. Although it is widely accepted that impaired fatty acids oxidation (FAO) play a crucial role in renal fibrosis progression, other lipid metabolic pathways, especially FAs uptake, has not been investigated in fibrotic kidney. In this study, we aim to explore the potential mechanically role of FAs transporter in the pathogenesis of renal fibrosis. In the present study, the unbiased gene expression studies showed that fatty acid transporter 2 (FATP2) was one of the predominant expressed FAs transport in TECs and its expression was tightly associated with the decline of renal function. Treatment of unilateral ureteral obstruction (UUO) kidneys and TGF-β induced TECs with FATP2 inhibitor (FATP2i) lipofermata restored the FAO activities and alleviated fibrotic responses both in vivo and in vitro. Moreover, the expression of profibrotic cytokines including TGF-β, connective tissue growth factor (CTGF), fibroblast growth factor (FGF), and platelet-derived growth factor subunit B (PDGFB) were all decreased in FATP2i-treated UUO kidneys. Mechanically, FATP2i can effectively attenuate cell apoptosis and endoplasmic reticulum (ER) stress induced by TGF-β treatment in cultured TECs. Taking together, these findings reveal that FATP2 elicits a profibrotic response to renal interstitial fibrosis by inducing lipid metabolic reprogramming including abnormal FAs uptake and defective FAO in TECs.

## Introduction

About 10% of general population have different degrees of kidney damage in the world. Kidney fibrosis, characterized by tubular atrophy, immune cells infiltration, and massive extracellular matrix deposition, is a final common pathological lesion in the progression of the chronic kidney disease (CKD)^1,2^. CKD causes high health cost and high mortality in the whole society because it would inevitably lead to end-stage renal failure (ESRD)^1^. At present, there is no effective method for treating renal fibrosis except dialysis and kidney transplantation in clinical, and it is of great significance to better understanding the pathogenesis of renal fibrosis so as to find the cellular and molecular regulator of this process, which may offer valuable insights for the development of effective preventing and therapeutic interventions.

Kidney is among the most energy-requiring organs in the body and the cytoplasm of renal tubular epithelial cells (TECs) contain amounts of mitochondria which is the core site for kidney energy metabolism^3^. Under physiological circumstances, TECs rely mainly on the β-oxidation of fatty acids (FAs) as their energy resource, which is similar to cardiomyocytes^4^. Under fibrotic conditions, defective fatty acids oxidation (FAO) as well as massive accumulated lipid is implicated in the altered lipid metabolism in TECs, leading to organ damage and renal dysfunction^3^. A previous study has shown that the expression of gene related to lipid metabolism pathway in patients with CKD is significantly reduce compared with the normal population by whole-genome sequencing^3^; the reduced expression of a series of FAO related enzymes and regulatory molecules is also observed in several mouse models of kidney fibrosis^3^. Chung^5^ et al. found that down-regulating the expression of peroxisome proliferator-activated receptor alpha (PPARα), one of key enzyme in β-oxidation pathway, significantly aggravates aging-related renal fibrosis progression. Further, it has been found that treatment with PPARα agonist fenofibrate can reverse TGF-β induced lipid deposition in TECs and improve tubulo-interstitial fibrosis and apoptosis;^3^ Specific overexpression of PPARγ coactivator-1a (PGC1α) in TECs also can partially restore β-oxidative function, effectively inhibit folate-induced renal fibrosis^3^. Several miRNAs have also been implicated in playing a crucial role in the pathogenesis of renal fibrosis by targeting key FAO transcriptional factors^6,7^. Therefore, rebalancing lipid metabolism by restore FAO activities may be a key point to prevent renal fibrosis development.

Intracellular lipid metabolism is precisely regulated by FAs biosynthesis, oxidation, uptake from the microenvironment, and their hydrolysis by lipid droplets (LDs) turnover. Although it is well established that deficiency in FAO is closely associated with the development of renal fibrosis^3^, whether other regulatory metabolic pathways participate in the progression of kidney fibrosis is still unknown and needs further investigated. An emerging evidence has shown that cluster of differentiation 36 (CD36), an important long-chain FAs transporter, is involved in the development of CKD, especially in the context of diabetic kidney diseases (DKDs), indicated a potential role of FAs uptake-related proteins in the pathogenesis of kidney fibrosis^8^. Intriguingly, although transgenic overexpression of tubular CD36 in fibrotic kidney led to an increased intrarenal lipid accumulation, these transgenic mice have not significantly exaggerated tubulo-interstitial fibrosis compared with wild-type mice^3^. Therefore, we speculate that other FAs transports, rather than CD36, may be a potential mediator of the fibrotic progress.

**Fig. 1:**
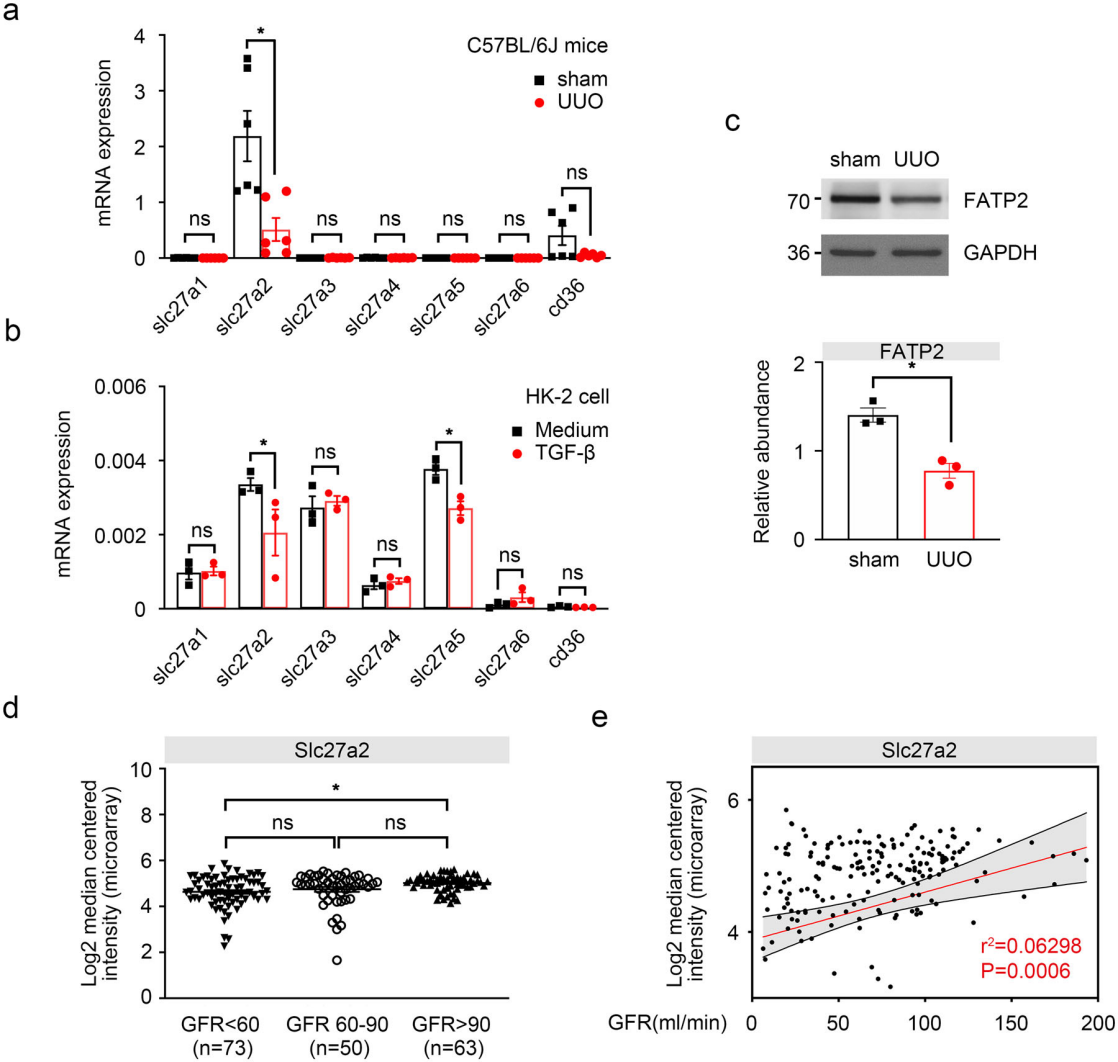
Renal tubular epithelial cells mainly expressed FATP2. **a** Relative transcript levels of FAs uptake-associated transporter in sham and UUO mouse models for 7 days. **b** Relative transcript levels of FAs uptake-associated transporter in HK-2 cells with 10 ng/mL TGF-β treatment for 48 h. **c** FATP2 and GAPDH protein expression by immunoblot analysis, GAPDH sets as loading control. Results were quantified using ImageJ software. **d** Nephroseq Analysis was conducted to evaluate the expression of FATP2 in human kidney biopsy sample. **e** CKD was associated with significantly reduced mRNA value of slc27a2 compared with biopsy samples from healthy control. Data are presented as mean ± SEM. *n* = 6 mice. *P < 0.05, ns means no statistical significance.

Our goal was to identify the candidate FAs transporter which is involved in the pathogenesis of kidney fibrosis, so that it could be serve as a promising therapeutic target to prevent CKD progression. In this study, we focus on fatty acid transport proteins (FATPs) family, another mostly mentioned proteins that are implicated in the uptake of lipids. Our exploratory studies revealed that FATP2 is one of the most abundant FAs transporters on unilateral ureteral obstruction (UUO)-induced fibrotic kidney and TGF-β induced TECs (Fig. 1a and Fig. 1b). Using the pharmacological inhibitory approach, we show that FATP2 regulates tubular lipid metabolism and could be the crucial inciting factor for kidney fibrosis development.

## Results

### FATP2 predominantly expressed in fibrotic kidney

Renal TECs are highly dependent on FAO to produce large amounts of ATP for normal function^3^. FAs, the main energy recourse for FAO, are absorbed from extracellular matrix through membrane proteins such as FATPs^9^ and CD36^8,10^. Quantitative RT-PCR analysis was performed to explore the expression profile of lipid transporters in TECs. Among FATPs family and CD36, FATP2 was the only highly expressed FAs transporter in kidney, which was consistent with previous studies. Although the mRNA level of FATP2 was considerable decreased in UUO-induced fibrotic kidney, its expression was still ranked at top among indicated FAs transporters. In addition, slightly expressed level of CD36 mRNA was detected in normal kidney but was markedly diminished in the UUO kidney (Fig. 1a). Similar to our observations in UUO mouse, slc27a2 (gene-encoded FATP2) was also one of the mainly expressed FAs transporters in human TECs line HK-2 cells, which was significantly reduced after TGF-β treatment (Fig. 1b). Surprisingly, we detected the high mRNA expression of slc27a5 in HK-2 cells; however, it was not found in the mouse kidney (Fig. 1a, b). These results were confirmed by densitometric analyses of the western blotting experiments (Fig. 1c). Next, Nephroseq Analysis (https://www.nephroseq.org/resource/login.html) was conducted to evaluate the expression of FATP2 in human kidney biopsy samples. In the study of Ju CKD tubules, CKD (eGFR less than 60 ml/min, *n* = 73) was associated with significantly reduced mRNA value of slc27a2 compared with biopsy samples from healthy control (eGFR more than 90 ml/min, *n* = 63) (Fig. 1d). Moreover, a positive correlation between tubular slc27a2 and eGFR was observed in public Ju CKD dataset (*r*^2^ = 0.0629, *p* = 0.0006, *n* = 186) (Fig. 1e), suggesting a potential role of FATP2 in the development of CKD. These data show that FATP2 as a fatty acid transport, it expression may be closely associated with CKD progression.

### Pharmacological inhibition of FATP2 by Lipofermata reduced lipid accumulation in TECs

**Fig. 2:**
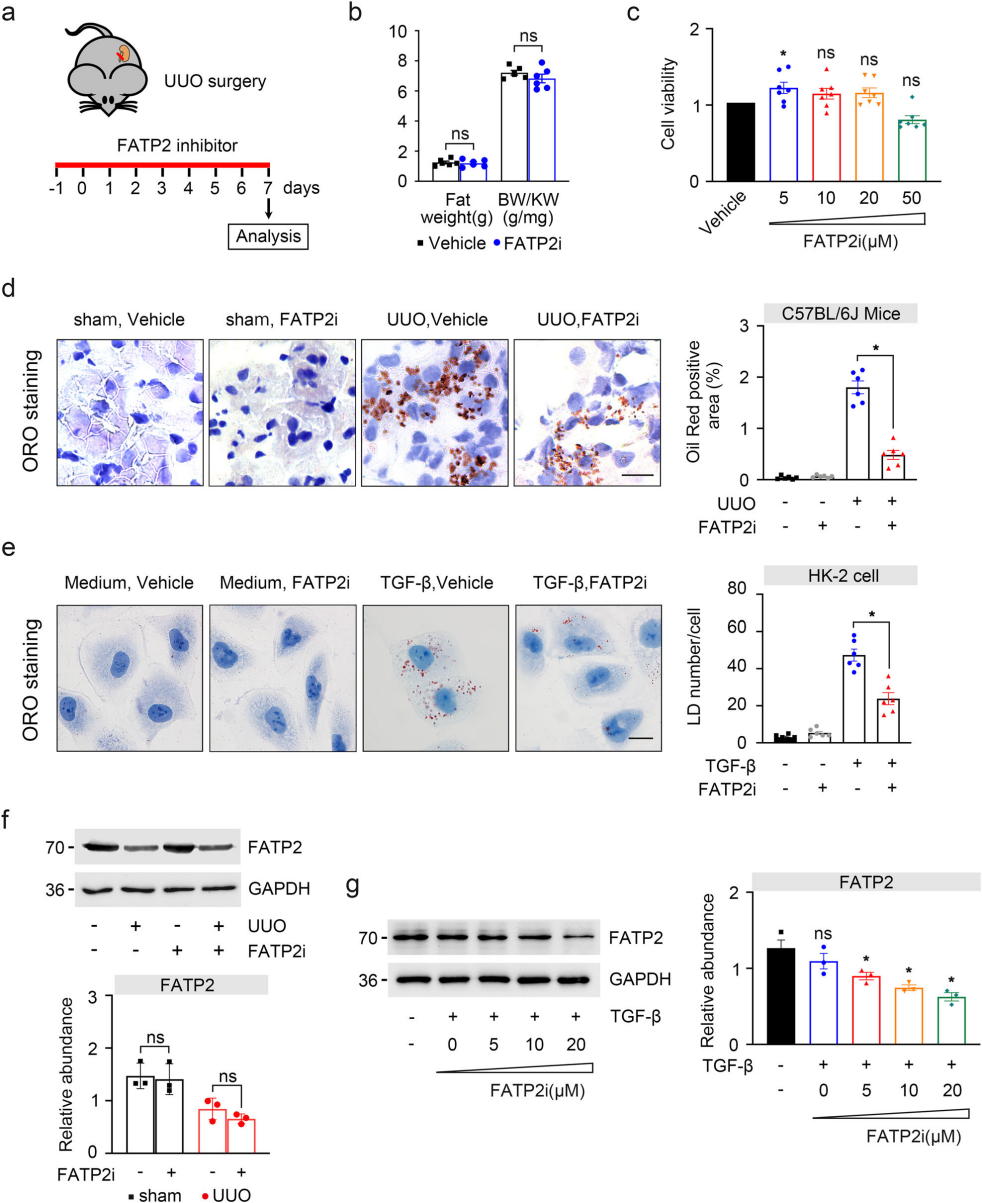
The relationship between FATP2 and intracellular lipid accumulation in fibrogenesis. **a** One day before UUO surgery treatment with FATP2 inhibitor lipofermata, and then sacrificed on day 7. **b** Effect of FATP2 inhibitors on fat weight and BW/KW ratio in mice (*n* = 6 mice per group). **c** Cell viability of HK-2 cell, which was treated by various doses of lipofermata (0, 5, 10, 20, 50 μM) for 48 h. **d** Oil red O staining detected the effects of FATP2 inhibitor on UUO mice fatty acids uptake. Schematic representation of oil red positive staining. Scale Bar: 50 μm. **e** Oil red O staining detected the effects of FATP2 inhibitor on TGF-β-induced HK-2 cells fatty acids uptake. Schematic representation of lipid droplet number. Scale Bar: 50 μm. **f**, **g** The kidney tissue and cell lysates were subjected to western-blot analysis with indicated antibodies against FATP2. Schematic representation of quantitative data of indicated proteins. Representative images from three independent experiments are shown above. *P < 0.05, ns means no statistical significance.

TECs lipid deposition has received extensive attention, especially in the context of acute and diabetic nephropathy^3,11^. It has been proposed that excess ectopic accumulation of triglycerides induces cellular lipotoxicity that may contribute to the development of renal fibrosis^12^. To further understand the role of FATP2 in tubular lipid metabolism during kidney fibrosis, lipofermata, a specific FATP2 inhibitor (FATP2i), was used in vivo model of UUO and in vitro model of TGF-β-treated TECs. Mice undergoing UUO were randomly treated with FATP2i or vehicle, and then sacrificed on day 7 (Fig. 2a). FATP2i administration did not change the fat weight of the UUO mouse and the ratio of BW/KW (Fig. 2b). Consistently with previous report, there was not obviously change in cell viability between control and FATP2i treatment by cell counting kit-8 (CCK8) assay, even in the dosage of 50 μM (Fig. 2c), indicated that lipofermata have few cytotoxic effects on tubular cells. Subsequently, the effect of FATP2 on transporting FAs was assessed by Oil Red O (ORO) staining. Most strikingly, UUO engagement dramatically increased tubular lipid accumulation, which was effectively prevented by FATP2i treatment (Fig. 2d), the same phenomenon has also been observed in TGF-β-treated TECs in vitro (Fig. 2e). In addition, the expression of FATP2 was determined by western blotting, FATP2 was relatively reduced in UUO mouse models and can be further decreased in FATP2i-treated kidney (Fig. 2f), these observations were verified in TGF-β induced HK-2 cells, as FATP2i effectively reduced the expression of FATP2 in a dose-dependent manner (Fig. 2g). These results indicate that pharmacological inhibition of FATP2 activity can attenuate lipid deposition in TECs.

### Pharmacological inhibition of FATP2 alleviated renal injury in UUO mouse models in vivo

**Fig. 3:**
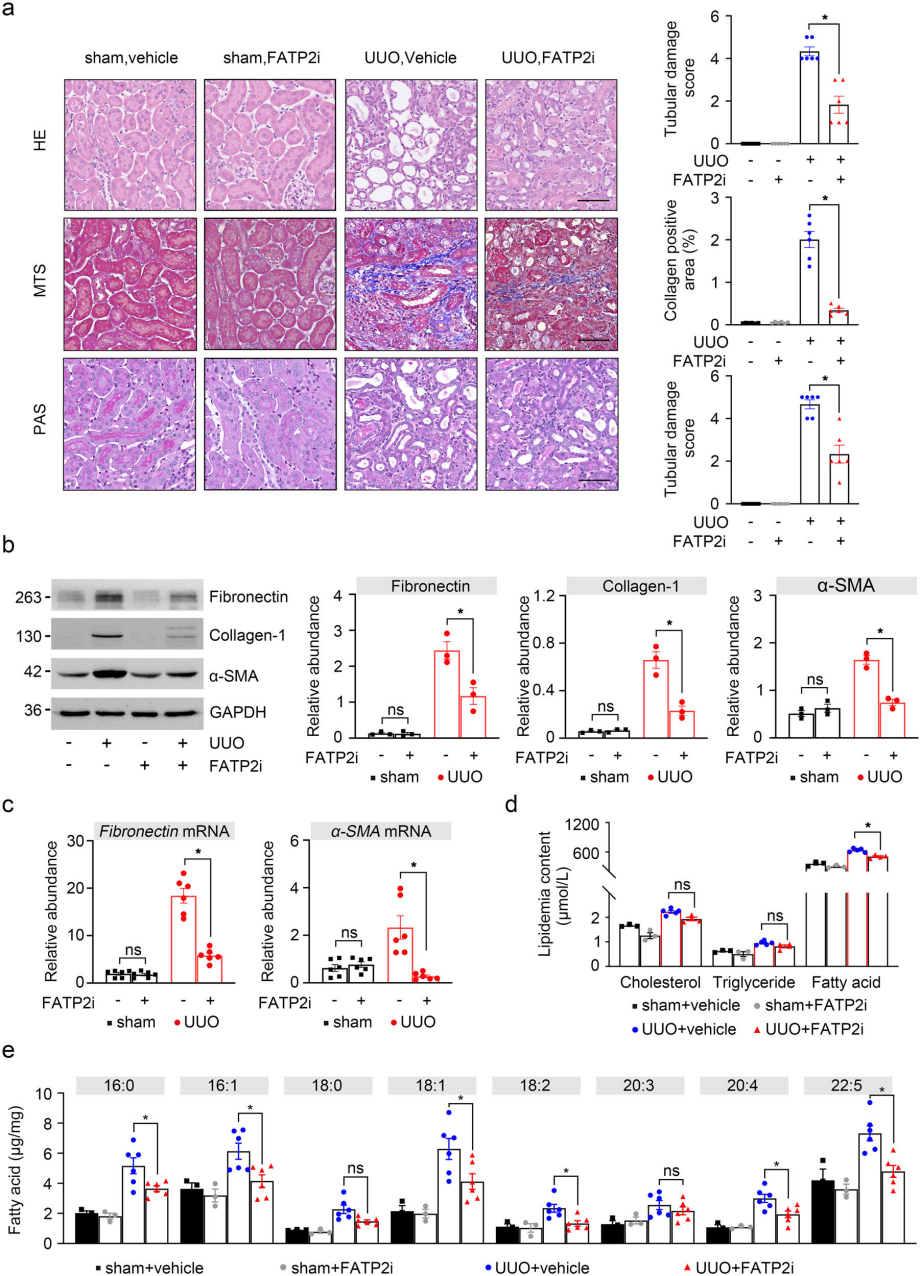
The effect of FATP2 inhibitor on fibrotic kidney. **a** Representative images of FATP2 inhibitor presence or absence in healthy and UUO mouse kidney sections stained with HE, MTS, PAS staining. Schematic representation of tubular damage score and collagen positive area. Scale Bar: 100 μm. **b** Fibronectin, collagen-1, α-SMA, and GAPDH protein expression by immunoblot analysis in mouse kidney. GAPDH sets as loading control. Schematic representation of quantitative data of indicated proteins. **c** Fibronectin and α-SMA mRNA expression by RT-qPCR analysis. **d** Contents of total cholesterol, triglycerides, and free fatty acids in peripheral blood of mice. **e** Total fatty acids in mouse kidney (mean ± SEM). *n* = 6 mice. *P < 0.05, ns means no statistical significance.

Based on the above experimental findings, we speculated whether inhibition of FATP2 activity contributes to renal fibrosis progression in vivo. UUO kidneys exhibited severe morphologic lesions characterized by tubular dilation, tubulo-interstitial expansion, tubular necrosis, and brush border loss (Fig. 3a). Interesting, these morphologic abnormalities were all ameliorated in the kidneys of FATP2i-engaged mouse (Fig. 3a). In addition, UUO-induced collagen deposition in the interstitium was also dramatically degraded in the present of FATP2i by Masson Trichrome staining (MTS) (Fig. 3a). FATP2i does not have a great effect on kidneys of healthy mice (Fig. 3a). By western-blot analysis, FATP2i-treated groups showed markedly lower levels of fibronectin, collagen I, and α-SMA than UUO groups (Fig. 3b). Consistent with the protein levels, the mRNA levels of fibronectin and α-SMA were also significantly reduced in FATP2i-treated kidney (Fig. 3c). We further measured the peripheral blood lipid content of animals, as shown in Fig. 3d, triglycerides, total cholesterol, free FAs were dramatically increased in UUO kidneys, but only fatty acid can be notably reduced by FATP2i treatment (Fig. 3d). We then investigated the role of FATP2 in regulating fatty acid by mouse kidney. Gas chromatography-mass spectrometry (GC-MS) analysis revealed a reduced amount of free fatty acid in UUO mice kidney after FATP2i treatment (Fig. 3e). FATP2i in the UUO mice resulted in a decrease in the total abundance of C16:0, C16:1, C18:1, C18:2, C20:4, and C22:5 (Fig. 3e). There are no significant changes were observed in C18:0 and C20:3(Fig. 3e). These findings suggest that inhibition of FATP2 may alleviate the development of renal fibrosis, partly through the inhibition of lipids transport.

### Pharmacological inhibition of FATP2 by Lipofermata alleviated renal fibrosis in HK-2 cells in vitro

**Fig. 4:**
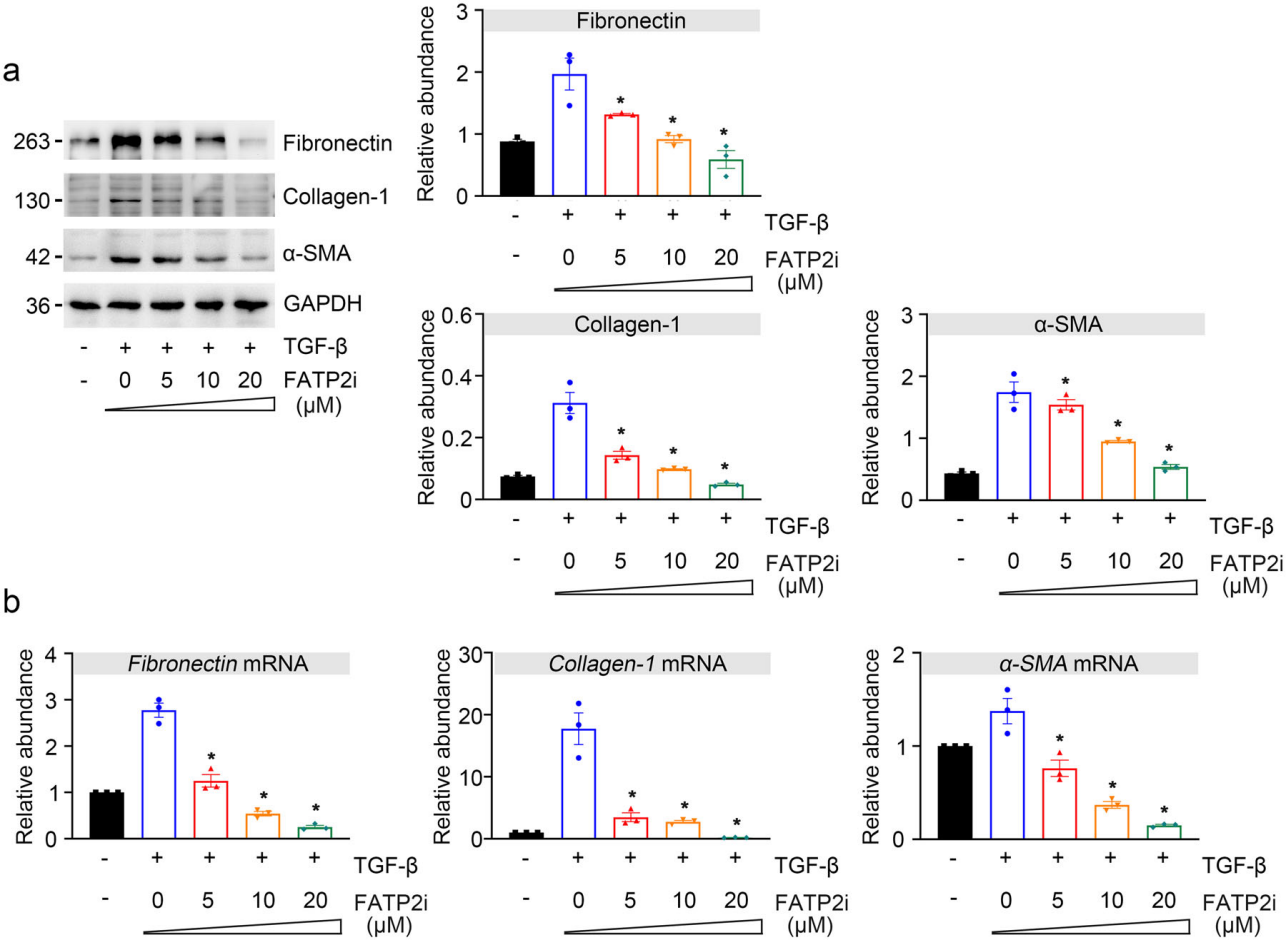
The effect of FATP2 inhibitor on TGF-β-induced HK-2 cell. **a** Fibrosis marker fibronectin, collagen-1, α-SMA, and GAPDH protein expression by immunoblot analysis in HK-2 cells. GAPDH sets as loading control. Schematic representation of quantitative data of indicated proteins. **b** Fibronectin, collagen-1, α-SMA mRNA expression by RT-qPCR analysis in HK-2 cells. These data are calculated from three independent experiments and are expressed as the mean ± SEM. *P < 0.05.

We further examined the role of FATP2 in TGFβ-induced profibrotic phenotype in TECs. In accordance with the results of in vivo experiments, the protein level of fibronectin, collagen I, and α-SMA was markedly increased in TGF-β-treatment groups than controls, and these fibrotic markers were all significantly reduced with used the specific-inhibitor of FATP2 in a dose-dependent manner (Fig. 4a). Similarly, compared with controls, gene expression for these fibrosis markers (fibronectin, collagen-1, and α-SMA) were all upregulated in TGF-β-treated TECs, and which is gradually downregulated after FATP2i treatment in a dose-dependent manner (Fig. 4b). Combined with the above-mentioned findings, our data indicate that FATP2 may be an initial factor that triggers off the development of renal fibrosis.

### FATP2 accelerates the development of fibrosis by upregulating profibrotic cytokines

**Fig. 5:**
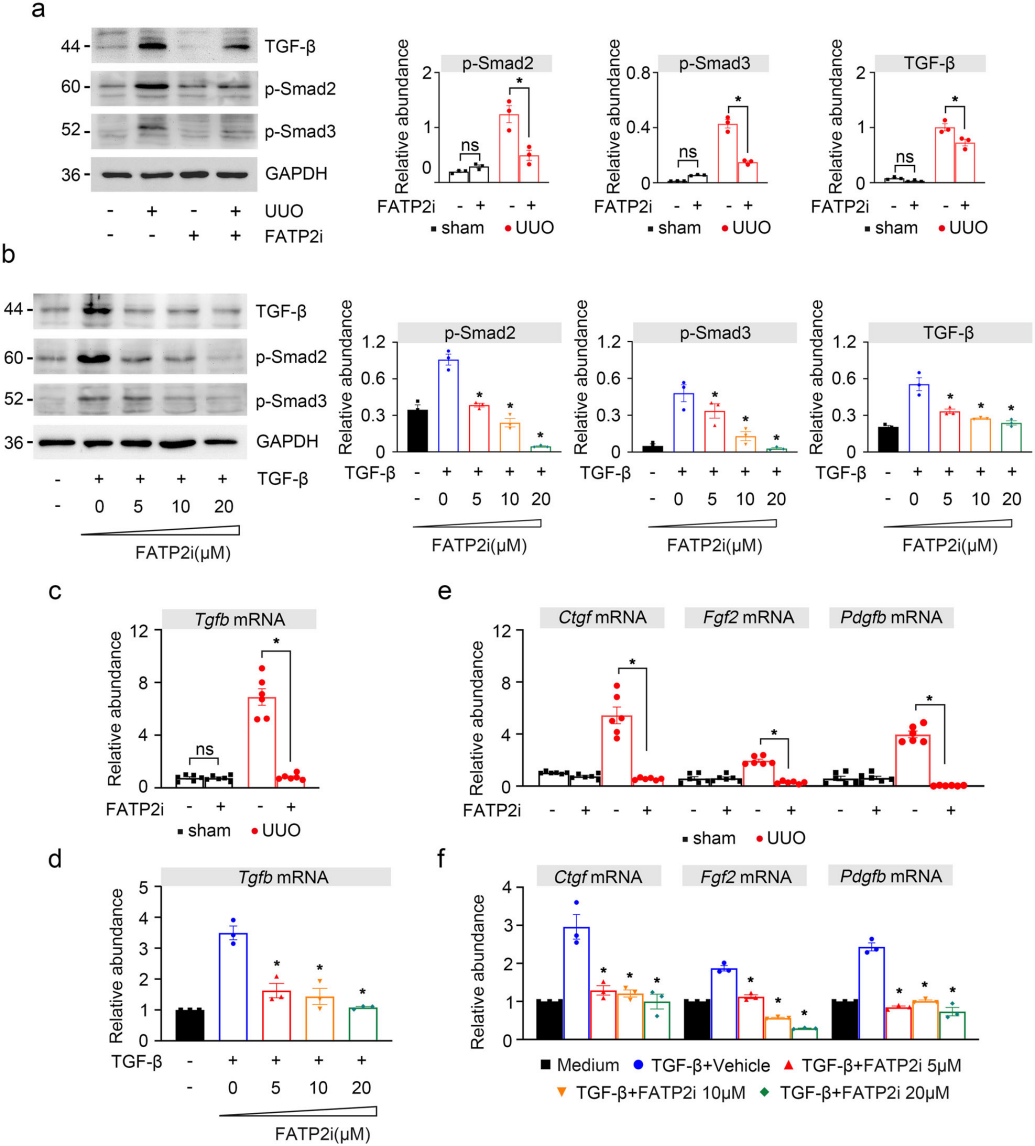
The effect of FATP2 inhibitor on profibrotic cytokines in vivo and in vitro. **a** TGF-β, p-Smad2, and p-Smad3 and GAPDH protein by immunoblot in UUO mouse samples. GAPDH sets as loading control. Schematic representation of quantitative data of indicated proteins. **b** TGF-β, p-Smad2, and p-Smad3and GAPDH protein by immunoblot in HK-2 cells. GAPDH sets as loading control. Schematic representation of quantitative data of indicated proteins. **c**, **e** Profibrotic cytokines TGF-β, Ctgf, Fgf2, Pdgfb mRNA expression by RT-qPCR analysis in UUO mouse. **d**, **f** Profibrotic cytokines TGF-β, Ctgf, Fgf2, Pdgfb mRNA expression by RT-qPCR analysis in HK-2 cells. Representative images from three independent experiments are shown above. *n* = 6 mice per group. *P < 0.05.

TGF-β is a profibrotic cytokine plays a major role in fibrogenesis^13^. It is well documented that TGF-β accelerates renal interstitial fibrosis by activating the Smad pathway to promote the production of myofibroblasts and the deposition of extracellular matrix^13^. PDGFB^14^, FGF2^15,16^ and connective tissue growth factor (CTGF)^17–19^ are also candidates profibrotic cytokines that contributes to renal fibrogenesis. To further determine the role of FATP2 in the development of renal fibrosis, we detected the relevant profibrotic factors. As shown in Fig. 5a, the treatment of UUO mice with FATP2i inhibited the expression of TGF-β by western-blot analysis and qPCR (Fig. 5a, c). Kidney expression of p-Smad2 and p-Smad3 were reduced in UUO mice following FATP2i treatment (Fig. 5a). Consistent with the results of experiments in vivo, FATP2i also notably reduced the expression of TGF-β and its associated downstream target protein of p-Smad2 and p-Smad3 in a dose-dependent manner in vitro (Fig. 5b). Moreover, PDGBB, FGF2, and CTGF transcripts were all blunted both in the UUO kidney (Fig. 5e) and in TGF-β-induced HK-2 cells after FATP2i treatment (Fig. 5d, f). Based on the above results, we speculate that, under fibrotic conditions, FATP2 may play a key role in promoting the progression of fibrosis by regulating the expression of profibrotic cytokines.

### Pharmacological inhibition of FATP2 by lipofermata restored FAO induced by UUO injury

**Fig. 6:**
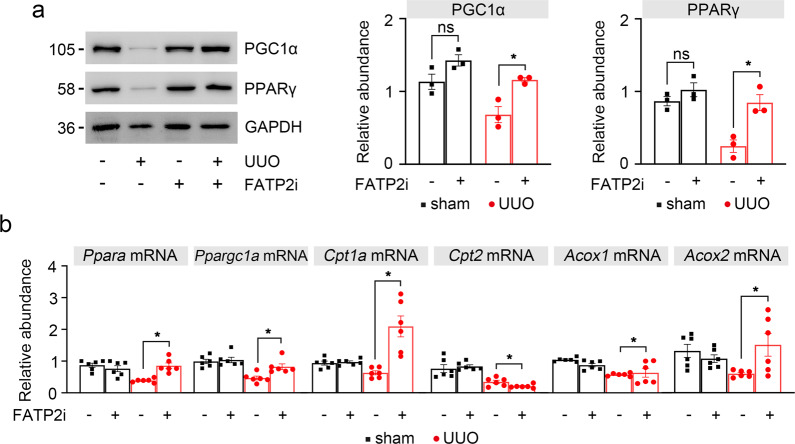
FATP2 inhibitor restored low expression of FAO key enzymes caused by fibrotic kidneys. **a** FAO key enzymes PGC1α and PPARγ protein expression by immunoblot analysis in UUO mouse kidney samples. GAPDH sets as loading control. Schematic representation of quantitative data of indicated proteins. **b** Relative mRNA levels of genes related to FAO in UUO mouse kidney samples by RT-qPCR analysis. These data are calculated from three independent experiments and are expressed as the mean ± SEM. *n* = 6 mice per group. *P < 0.05, ns means no statistical significance.

Healthy renal TECs rely primarily on FAO as their energy source^3^. Previous study indicated that impaired FAO in TECs contributes the development of tubulo-interstitial fibrosis^3^. Of note, the peroxisome proliferator-activated receptors (PPARs) and PPAR-γ coactivator-1α (PGC1α) are the key transcription factors involved in fatty acid uptake and oxidation^3,20^. PPARs and PGC1α can alter β-oxidation progress of fatty acids by regulating the activities of multiple FAO downstream key enzymes, such as carnitine palmitoyltransferase-1 α (Cpt1α), carnitine palmitoyltransferase-2 (Cpt2), and acyl-CoA oxidase (Acox)^20^. So far the role of FATP2 involved in FAO during renal fibrosis has not been explored. Western blotting analysis showed that the expression of PGC1α and PPARγ were significantly increased in UUO mice by FATP2i treatment (Fig. 6a). Consistent with immunoblot analysis, results of quantitative PCR shown that FATP2i normalized UUO-induced defective FAO, as indicated by the restored transcript levels of key and rate-limiting enzymes of FAO (Ppara, Ppargc1a, Cpt1, Cpt2, Acox1, and Acox2) (Fig. 6b). These data suggest that inhibition of FATP2 may rehabilitate impaired tubular FAO in UUO-induced fibrotic kidney.

### FATP2 inhibition protects against TGF-β induced apoptosis and endoplasmic reticulum (ER) stress

**Fig. 7:**
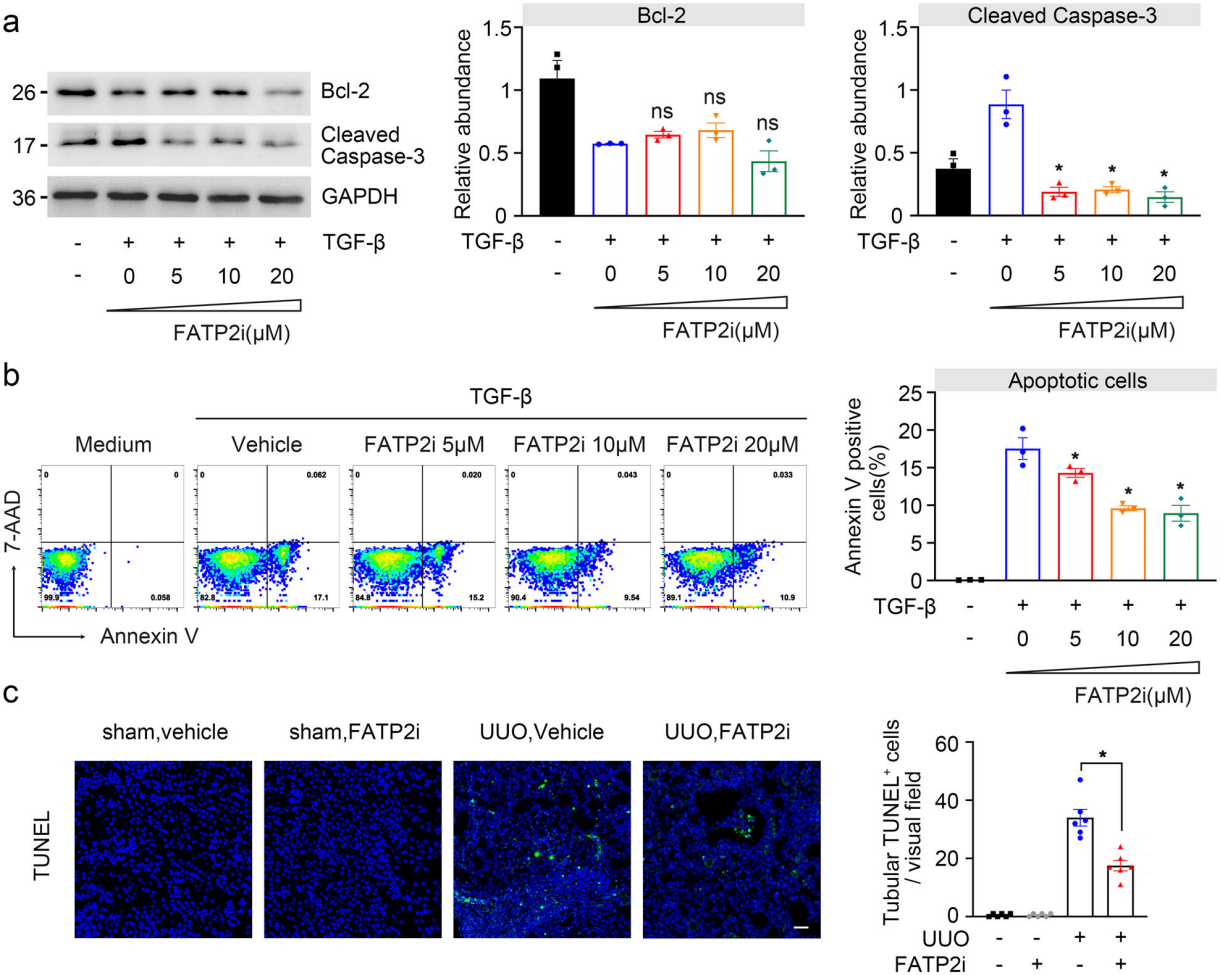
FATP2 inhibitor reduced lipoapotosis caused by fibrotic kidneys. **a** Bcl-2 and Cleaved caspase 3 protein expression by immunoblot analysis in HK-2 cells. GAPDH sets as loading control. Schematic representation of quantitative data of indicated proteins. **b** FATP2 inhibitor decreased apoptosis by flow cytometry caused by TGF-β induced fibrogenesis. **c** TUNEL staining of kidney tissues (green: TUNEL-positive cells, blue: DAPI, x200). These data are calculated from three independent experiments and are expressed as the mean ± SEM. *P < 0.05, ns means no statistical significance.

Disturbed lipid metabolism-associated lipotoxicity is characterized by a series of events including ER dysfunction and initiation of the apoptotic death program, which are contributed to the development of renal fibrosis^21–23^. To investigate whether FATP2 inhibitor was effective in protecting against lipotoxicity in vitro model of TGF-β-treated TECs, we incubated HK-2 cells with TGF-β in the absence or presence of FATP2i for 48 h. TGF-β stimulation markedly reduced Bcl-2 level and increased cleaved caspase 3 level compared with vehicle-treated cells. These changes were ameliorated by pretreatment with FATP2 inhibitor (Fig. 7a). Because Bcl-2 is a negative mediator of cell apoptosis, while levels of cleaved caspase 3 are positive associated with apoptotic activities, these data suggest that FATP2i may mitigate TGF-β initiated cell apoptosis. Flow cytometric analysis further confirmed our findings as FATP2i considerably diminished annexin V-positive cells in TGF-β treated TECS in a dose-dependent manner (Fig. 7b). In vivo model of UUO mice, we analyzed the apoptosis by the terminal deoxynucleotidyl transferase-mediated dUTP nick-end labeling (TUNEL). A large number of TUNEL-positive cells was observed in the UUO-injured kidney and FATP2i administration diminished the number of TUNEL-positive cells (Fig. 7c).

**Fig. 8:**
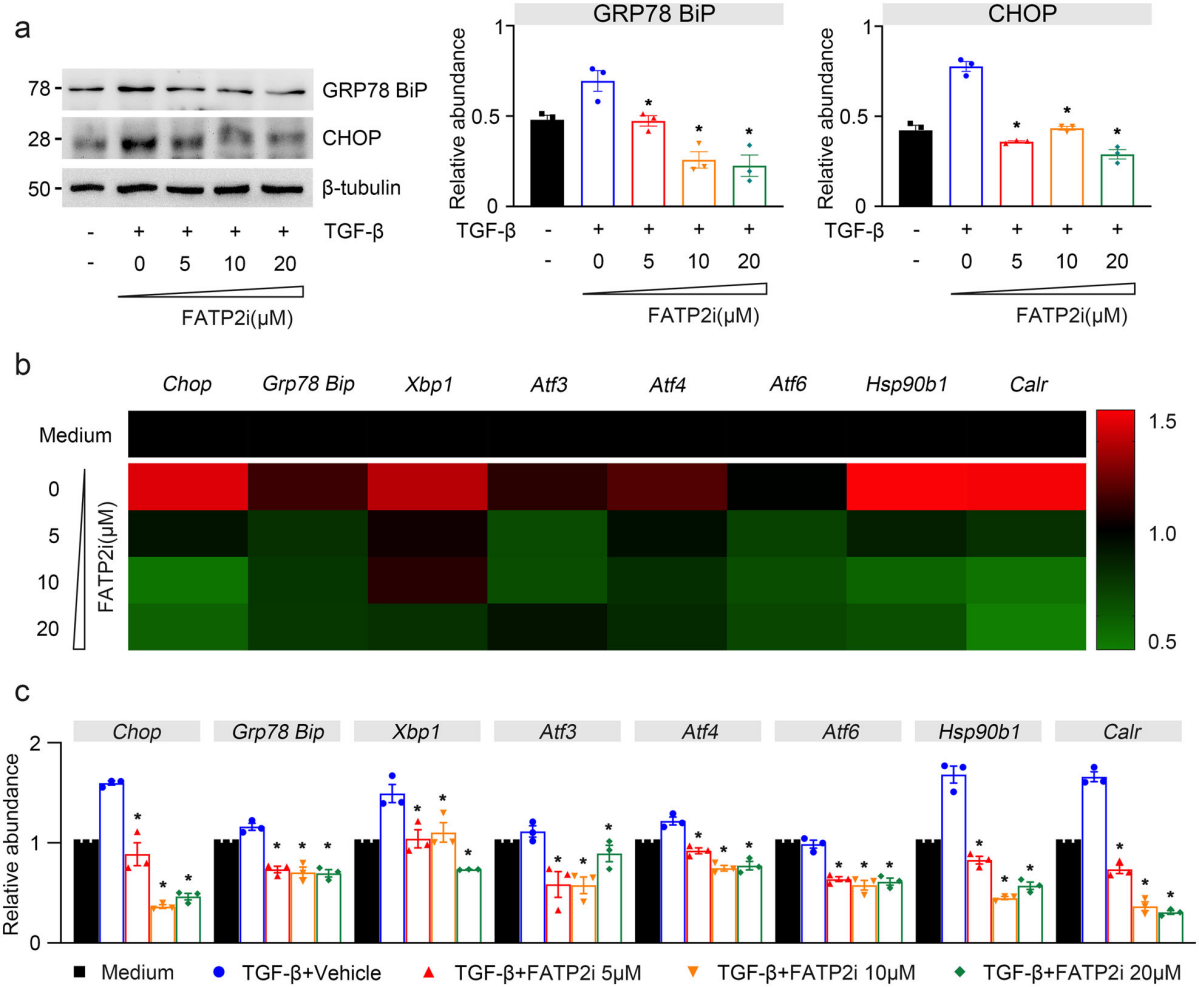
FATP2 inhibitor reduced ER stress caused by fibrotic kidneys. **a** ER stress related indicator BIP and CHOP protein expression by immunoblot analysis in HK-2 cells. GAPDH sets as loading control. Schematic representation of quantitative data of indicated proteins. **b** Relative mRNA levels of genes related to ER stress in TGF-β-treated TECs by RT-qPCR analysis. These data are calculated from three independent experiments and are expressed as the mean ± SEM. *P < 0.05.

ER is a multifunctional organelle that coordinates protein folding, lipid biosynthesis, calcium storage, and release. Perturbations of ER homeostasis lead to ER stress and activation of signaling cascades termed the unfolded protein response (UPR)^24^. Excessive ER stress can activate UPR-dependent apoptotic death^24^. To assess the relationship between FATP2 and ER stress, we first tested the expression of ER stress-associated proteins under different dosage of FATP2i treatment. As shown in Fig. 8a, BIP and CHOP, two important UPR molecules responsible for ER stress induction, were markedly evaluated in TGF-β-treated TECs and can be notably inhibited by FATP2i (Fig. 8a). Moreover, multiple UPR targets genes, including Chop, Grp78, Xbp1, Atf3, Atf4, Atf6, Hsp90b1, and Calr, were all substantially mitigated after FATP2i treatment in TGF-β stimulated HK-2 cells (Fig. 8b, c). Taken together, these data indicate that FATP2 may represent a cell injury mechanism that contributes to cell apoptosis and ER stress during the renal fibrogenesis in vitro experimental model.

## Discussion

During kidney fibrosis, TECs undergo significant metabolic changes, but the mechanism of how abnormal lipid metabolism affects fibrosis development have been unclear. Here we find that, FATP2 plays an important role in renal fibrosis, it accelerates fibrosis progression by reprogramming lipid metabolism, up-regulates the expression of fibrotic cytokines, and leads to cell injury by inducing apoptosis and ER stress.

FATP2 is a member of the Slc27a (solute carrier family 27) family and mainly mediates the uptake of exogenous FAs^25,26^. Consistent with earlier reports, our study has also shown that FATP2, but not CD36, is the candidate transporter that regulates FAs absorption in kidney^9^. CD36 has been considered to be a major FA transporter and important player in FA accumulation^8,10^. However, recent studies have shown that FATP2 is mainly expressed in the proximal tubule, while CD36 is expressed in renal macrophages, but not in the proximal tubule in mice^9^. Lipid homeostasis are precisely regulated by diverse molecules, the regulation of lipid metabolism in TECs relies on CD36-independent pathway, therefore highlights the key role of FATP2 in regulating lipid homeostasis in TECs.

Surprisingly, our data showed that FATP2 expression is slightly reduced during fibrosis, as well as a large number of LDs are accumulated in TECs. This inconsistent phenomenon may due to the different executive functions of FATP2 isoforms. FATP2 has two splice variants, FATP2a and FATP2b, which are localized in the cell membrane and peroxisome, respectively^26^. Both splice variants participate in the uptake and transport of exogenous FA, however, only the former exerts acyl-CoA synthetase activity, which mediates vectorial acylation of long-chain FAs^26^. Only FATP2a has been detected in kidney tissue^9^. Perez^27^ et al. found that genes encoding fatty acid-binding protein 1 (Fabp1), CD36 and the long-chain acyl-CoA synthetase (Acsl1) were remarkably increased expression in the Fatp2^−/−^ mice liver transcriptome. It is reasonable to consider that these Fabps might also play a role in kidney fibrosis.

Lipofermata is identified as a specific inhibitor of FATP2, which alleviates lipoapoptosis and cell death through mediating the uptake of long-chain FAs^25^. Lipofermata can degrade triglyceride in the plasma of acute kidney injury mouse models, and obviously improve lipid deposition in palmitate acid-treated various cell lines^23^. Our data indicate that FATP2i can significantly mitigate intracellular lipid accumulation and delay the progression of renal fibrosis. A previous study has shown that compared with wild-type mice, Slc27a2−/− mice manifested that slighter tubular atrophy and interstitial fibrosis in various acute kidney injury mouse models^23^. Additionally, compared with wild-type mice, interstitial fibrosis and renal tubular damage were significantly reduced in treatment Slc27a2−/− mice with zoledronate^28^.

Most strikingly, in our study, we found that FATP2 can regulate the progression of renal fibrosis by mediating the secretion of several profibrotic cytokines (TGF-β, CTGF, PDGFB, FGF2), but the specific mechanism remained unclear. CTGF^19,29,30^, PDGFB^14^, and FGF2^31^ have been demonstrated the important inducers which are involved in the pathogenesis of epithelial-mesenchymal transition (EMT). All of these are candidate factors that are considered to mediate the downstream profibrotic action of TGF-β^18,32,33^. CTGF overexpression in kidney is associated with cell and extracellular matrix (ECM), which has been confirmed in human biopsies of various kidney diseases and in different experimental models of renal lesions^34,35^. Epidermal growth factor receptor (EGFR) can be combined with CTGF and its C-terminal degradation product CCN2(IV) to regulate inflammation and ECM production^36–38^. Inhibiting CTGF with antisense oligonucleotides or gene silencing was beneficial for experimental renal damage models, such as UUO mouse models, DN, and uninephrectomized TGF-β transgenic mice^17,39–41^. PDGFB mainly acts as a mitogen for the phenotype of myofibroblast, promotes mesangial cells proliferation and recruitment, and aggravates kidney lesion during fibrosis^14^. CTGF binds to PDGF receptor (PDGFR), leading PDGFR to be phosphorylated, and activating various cytoplasmic downstream signaling pathway, for instance, rat sarcoma-mitogen-activated protein kinase (Ras-MAPK), phosphatidyl-inositol-3-kinase (PI3K), phospholipase C-γ (PLC-γ) pathway and others^14^. FGF2 promotes renal fibroblasts proliferation, and its expression is significantly increased during fibrosis^15,31^. FGF2 executes its function by binding to and activating fibroblast growth factor (FGF) receptors, particularly FGFR1, to regulate renal fibrogenesis^42^. A previous study has shown that Klotho could inhibit the action of FGF2 by suppressing extracellular signal-regulated protein kinase 1/2 activation^16^. Tracks of Smad2/3 ChIP-seq are shown that the SMAD3 peak in the promoter region of the slc27a2 gene^28,43^. TGF-β1 may potentially regulate slc27a2 transcript levels directly through activated TGFβ-Smad2/3 signaling^28^.

Renal TECs are one of the most metabolic active cells in human body, which primarily depends on fatty acid oxidation to provide energy for a kidney^3^. Previous studies and our data both confirmed that defective FAO and excessive uptake of FAs lead to lipid accumulation, treatment with FATP2i can restore the progress of FAO and improve lipotoxicity in UUO mice^3^. Of note, PPARα is widely distributed in many metabolic organs, such as heart, liver, kidney, and adipose tissue^20^. In kidney, it mainly expressed in proximal tubules. FATPs are direct PPARα target genes, which catalyses ATP-dependent esterification of FAs into acyl-CoA derivatives^44,45^. Recently, study has shown that SLC27A2 ablation can restore FAO-related key enzymes activity caused by kidney damage, and protect TECs from lipotoxicity^28^. Deleting FATP2 in the mouse liver also found it changes the metabolic landscape by increasing the expression of PPARα^27^. Interestingly, PPAR signaling pathway can be downregulated by TGF-β in almost all fibrotic tissues^46–48^. A previous study shows that FATP2 is not selective transporter for arachidonic acid, its overexpression in polymorhonuclear myeloid-derived suppressor cells (PMN-MDSCs) favors increased uptake and trafficking of the fatty acid^49^. Transcriptome analysis identified changes in genes encoding enzymes involved in both arachidonic acid in FATP2^−/−^ mouse liver^27^. Therefore, we imply that FATP2 is not only a fatty acid transporter, but a momentous regulator promotes the development fibrosis by activating TGF-β and down-regulating PPARα, and both of which inhibit Ppargc1a expression and its downstream molecules. However, the specific mechanism remains to be further studied.

Recent studies have demonstrated that the UPR sensors directly involved in the regulation of lipid metabolism, and lipotoxicity can activate an ER stress response in liver^24^. In our study, inhibition of FATP2 activity markedly decreased UPR, it is shown that FATP2 regulates lipid metabolism in TECs, subsequently activates UPR, and mediates TECs apoptosis. Current studies have confirmed that lipofermata can alimented apoptosis and ER stress in palmitate acid-treated HepG2, INS-1E, and HRPT cells^23^. Disorders of lipid metabolism in renal TECs are closely related to the progress of CKD. FATP2 is a key molecule involved in lipid droplet uptake. Inhibition of FATP2 activity attenuates intracellular lipid aggregation lipotoxicity, alleviates the development of renal fibrosis, and it was clearly demonstrated its potential as a therapeutic to address lipotoxic disease.

In conclusion, our findings decipher an unexpected role of FATP2 in tubular lipid metabolism by inducing excessive FAs uptake and impaired FAO activities during kidney fibrosis. In addition, we also found that inhibition of FATP2 ameliorate kidney fibrosis by regulating profibrotic cytokine secretion, apoptosis, and ER stress. This study demonstrated that FATP2 might act as a promising candidate for the prevention of kidney fibrosis.

## Materials and methods

### Antibody and reagents

Anti-FATP2 (Santa Cruz, sc-393906, WB, 1:2000), anti-Fibronectin (Abcam, ab45688, WB, 1:5000), anti-α-SMA (Abcam, ab124964, WB, 1:10000), anti-Collagen I (Proteintech, 14695-1-AP, WB, 1:1000), anti-TGF-β1 (Proteintech, 21898-1-AP, WB, 1:1000), anti-p-Smad2 (CST, 3104S, WB, 1:1000), anti-p-Smad3 (CST, 9520T, WB, 1:1000), anti-PGC1α (Abcam, ab54481, WB, 1:1000), anti-PPARγ (Proteintech, 16643-1-AP, WB, 1:1000), anti-caspase 3 (Proteintech, 19677-1-AP, WB, 1:1000), anti-Bcl-2 (Santa Cruz, sc-7382, WB, 1:1000), anti-BIP (CST, 3177 S, WB, 1:1000), anti-CHOP (CST, 2895 T, WB, 1:1000). Recombinant human TGF-β1 (Peprotech, 100-21C-10), Oil Red O (Sigma-Aldrich, O0625). FATP2 inhibitor lipofermata (ChemBridge, 5830995).

### Animals

Male 8–12-week C57BL/6 mice were used in these experiments. Animals were house in a specific pathogen-free (SPF) environment in the laboratory animal center of Tongji hospital of Tongji Medical College. All mice handling and experimentation was conducted according to the guidelines of the National Health and Medical Research Council of China. All animal studies were reviewed and approved by the animal ethics review board of Tongji hospital of Tongji Medical College.

Mice were fed ad lib with normal diet during the whole experimental period. For the UUO model, the mice were first intraperitoneally injected with 1% pentobarbital sodium, and subsequently the left ureter was exposed and isolated following mid-abdominal incision. For UUO, the mid-ureter was obstructed using 2-point ligations with silk sutures (4-0), and sham-operated mice underwent the same procedure without the obstruction of the left ureter. After surgery, the incision was closed, and the mice were bred for indicated time points prior to euthanization. The kidney tissues were collected for further experiments.

For assess inhibition of FATP2, the mice were treated with lipofermata (50 mg/kg/day) in flaxseed oil at 1 h prior to surgery and continuously received daily for UUO duration. Controls received a vehicle consisted of flaxseed oil alone. The mice were sacrificed on day 7 after surgery, and the kidneys were obtained for further analysis.

### Cell culture

The human tubular cell line HK-2 was purchased from China Centre for Type Culture Collection (CCTCC, China) were maintained in DMEM-F12 (HyClone) medium plus 10% fetal bovine serum (FBS) and 1% penicillin-streptomycin in a 95% air 5% CO_2_ atmosphere at 37 °C. At 80% confluence, cells were treated with 10 ng/mL TGF-β1 with or without lipofermata for 48 h.

### CCK-8 assay

Cell viability assay was measured by Cell Counting Kit-8 (Bimake, China). HK-2 cells were cultured at 1 × 10^4^ cells per well in a 96-well plate, when reaching 80–90% confluence, cells were treated with lipofermata for 24 h. Lipofermata were diluted to obtain different concentration gradients. Absorbance was detected at 450 nm after treatment with 10 μL of CCK8 reagent for 2 h. The experiments were performed with six replicated wells per sample, and the assays were conducted in triplicate.

### Histological analysis

Kidney samples fixed in 4% paraformaldehyde and embedded in paraffin were stained with hematoxylin-eosin (HE), periodic acid-Schiff (PAS), and MTS according to the manufacturer’s instructions. The renal tubular damage score was based on tubular necrosis grade, cast formation, tubular dilation, and brush border loss, with scores corresponding to the following percentages of renal tubular damage: 0, 0%; 1, ≤10%, 2, 11 to 25%; 3, 26 to 45%; 4, 46 to 75%; and 5, ≥76%. The mean score of each sample was compared. The samples were examined using microscope (Mshot, China), and the images were analyzed using ImageJ software (National Institutes of Health, Bethesda, MD).

### Oil red O staining

Frozen sections (10 μm) of kidney tissues or cell coverslips were first washed in PBS and subsequently fixed with 4% paraformaldehyde for 20 min, washed three times in PBS, incubated in 60% isopropyl alcohol for 10 s, followed by staining with freshly prepared 60% Oil Red O solution (100% solution: 0.5 g of Oil Red O dissolved in 100 mL of isopropylene) for 30 min and washing with PBS. The samples were counterstained with hematoxylin. The slides were visualized using a Mshot microscope (Mshot, China). Staining was quantified using ImageJ software following standard protocols.

### Quantitative RT-PCR

Total RNA isolated from harvested cell lines and mouse kidney cortex using the Hipure Total RNA Mini Kit (R4111-03, Magen, China) according to the manufacturer’s instructions. RNA concentrations were determined using the NanoDrop 2000 Spectrophotometer (Thermo Fisher Scientific). cDNA was generated with a ReverTra Ace qPCR RT kit (FSQ-101, Toyobo, Japan). RT-qPCR was performed using an ABI Step One Plus system (Applied Biosystems, USA) with SYBY Green PCR mix (QPK-201, Toyobo, Japan) in 96-well plates. The data were normalized and analyzed using the 2^−^^ΔΔCt^ method. The primers used are listed in Supplementary Table 1.

### Western blotting

Cells or kidney tissues were lysed in RIPA buffer in the presence of 1% PMSF and 1% protease inhibitor cocktail, sonicated and stored at –80 °C. Protein concentrations were measured by BCA assay. Whole-cell lysates were loaded into the wells of 10% SDS-PAGE gel with equal amounts and subjected to electrophoresis, and then transferred to PVDF membrane (Millipore, USA) and blocked in 5% skim milk for 1 h at room temperature. Subsequently, the membranes incubated with indicated primary antibodies and following secondary antibodies. UVP imaging system (UVP, USA) was used to scan the membranes and Quantity One analysis software was applied to quantify the band intensity of blotted proteins.

### Total fatty acids analysis

A total of mouse kidney tissue from four groups were obtained for the metabolomics study using the GC-MS technique. Six biological replicates were used per sample. Samples were placed on ice and homogenized in 3 mL of chloroform/methanol (2:1, v/v) containing 0.05% butylated hydroxytoluene, then centrifuged at 13,000 rpm at 4 °C for 15 min. The supernatant was vortexed for 30 s and centrifuged at 12,000 rpm for 5 min. And then the samples dried under nitrogen gas and stored at −20 °C for further GC-MS analysis. Samples were reconstituted in isopropanol/acetonitrile (1:1) at 100 μL per samples prior to the GC-MS analysis.

### Flow cytometry

Cell apoptosis was performed using BD Annexin V apoptosis detection kit (556547) according to the manufacturer’s instructions. In brief, cells were collected and washed twice with ice-cold PBS, then incubated with indicated fluorescent dyes at 4 °C for 20 min. Cells were run on AccuriC6 (BD Biosciences) and data were analyzed by FlowJo software (Tree Star, Ashland, OR).

### TUNEL assay

The terminal deoxynucleotidyl transferase-mediated dUTP nick-end labeling (TUNEL) staining was conducted on paraffin-embedded slides using the in situ Cell Death Detection Kit (Roche, Indianapolis, IN) according to the manufacturer’s instruction. The tissue sections were detected using confocal laser microscopy (Olympus FV1200, Tokyo, Japan), and the images were analyzed using Image J software.

### Statistical analyses

All data were expressed at means ± SEM. Statistical analysis was performed using GraphPad Prism 8 (GraphPad Software Inc. San Diego, CA). For data analyses with one variable, one-way ANOVA multiple comparison was used, and 2-tailed unpaired or paired Student’s *t* test analysis was used for two-group comparisons. Two-way ANOVA test with Bonferroni correction for multiple comparisons. *P* values < 0.05 were considered statistically significant.

## Supplementary information

Supplementary Table 1
