# Supplementary material for: Involvement of FATP2-mediated tubular lipid metabolic reprogramming in renal fibrogenesis
Source: Cell Death Dis. 2020 Nov 20;11(11):994. doi: 10.1038/s41419-020-03199-x (PMC7679409; doi:10.1038/s41419-020-03199-x)
Supplement: Supplementary file 1 — Supplementary Table 1 [file 41419_2020_3199_MOESM1_ESM.docx]

**Supplementary Date**

**Table 1**

| Gene | Forward (5’→3’) | Reverse (5’→3’) |
| --- | --- | --- |
| SLC27A1 | GGTCGGCTCCTGTGGTTTCAAC | GCAGCAGCTCCATTGTGTCCTC |
| SLC27A 2 | GGAGATACATTCCGGTGGAA | TGATCTCAATGGTGTCCTGT |
| SLC27A 3 | GGCTGAAGTGTCCGCTGAAGTG | GGCTGAAGTGTCCGCTGAAGTG |
| SLC27A 4 | CTGTTGTTCCTCTACTTGGGAT | GTCTCGGAAGTACAGGTAGC |
| SLC27A 5 | CACGTTCAAACTGATGAAGACC | CCAGTACAAACAGAGGGTCAA |
| SLC27A 6 | TCATAGTTCAGCAGCTATCCTG | ATACTTCTTGCAGTCACTCCAA |
| CD36 | CTGTTATGGGGCTATAGGGATC | ACTCCATCTGCAGTATTGTTGT |
| FN | AATAGATGCAACGATCAGGACA | GCAGGTTTCCTCGATTATCCTT |
| COL1A1 | AAAGATGGACTCAACGGTCTC | CATCGTGAGCCTTCTCTTGAG |
| ACTA2 | CTCTGGACGCACAACTGGCATC | GGCATGGGGCAAGGCATAGC |
| TGFB1 | CTGTACATTGACTTCCGCAAG | TGTCCAGGCTCCAAATGTAG |
| FGF2 | CATCAAGCTACAACTTCAAGCA | CCGTAACACATTTAGAAGCCAG |
| PDGFB | GATCCGCTCCTTTGATGATCTC | GGTCATGTTCAGGTCCAACTC |
| CTGF | ATTCTGTGGAGTATGTACCGAC | GTCTCCGTACATCTTCCTGTAG |
| CHOP | GAGAATGAAAGGAAAGTGGCAC | ATTCACCATTCGGTCAATCAGA |
| BIP | CAGTTGTTACTGTACCAGCCTA | CATTTAGGCCAGCAATAGTTCC |
| ATF3 | TAGCCCCTGAAGAAGATGAAAG | CTTCTTCTTGTTTCGGCACTTT |
| ATF4 | ATGGATTTGAAGGAGTTCGACT | AGAGATCACAAGTGTCATCCAA |
| XBP1 | CTTGTAGTTGAGAACCAGGAGT | CCCAACAGGATATCAGACTCTG |
| HSP90B1 | TCTGAATTGATTGGCCAGTTTG | GGGTATCGTTGTTGTGTTTTGA |
| CALR | AGATAAAGGTTTGCAGACAAGC | CATGTCTGTCTGGTCCAAACTA |
| GAPDH | ACCAAATCCGTTGACTCCGAC | CTCCTGTTCGACAGTCAGCC |
| Slc27a1 | CCTCTCTGTTCTGATTCGTGTT | GTCCAGCATATACCACTACTGG |
| Slc27a2 | CCCAGGATGTCATCTATACCAC | CAATGTACTGAATGACCGTGAC |
| Slc27a3 | GTGACAGTGTTCCAGTACATTG | TCCTGTGTAATTGAACGTAGCT |
| Slc27a4 | GGTTACCTGTACTTCCGAGATC | CCTTTTTCAAGGTCTGTGCAAA |
| Slc27a5 | AGGAGCAGCTCTTACCTTATTC | GTGAAAGCCACATCTTTATGCA |
| Slc27a 6 | CTGGTCACGGTGCTGGATAAGTTC | AGCGAGGAGTGGTTCAGGAGAG |
| Cd36 | CTTTGAAAGAACTCTTGTGGGG | GTCTGTGCCATTAATCATGTCG |
| Fn | ACAGTCCAGCAAGCAGCAAGC | TGGTGGTCACTCTGTAGCCTGTC |
| Acta2 | GGCTTCGCTGGTGATGATGCTC | TCCCTCTCTTGCTCTGGGCTTC |
| Tgfb1 | CCAGATCCTGTCCAAACTAAGG | CTCTTTAGCATAGTAGTCCGCT |
| Fgf2 | AGTTGTGTCTATCAAGGGAGTG | CATTGGAAGAAACAGTATGGCC |
| Pdgfb | GTCCAGGTGAGAAAGATTGAGA | GTCATGGGTGTGCTTAAACTTT |
| Ctgf | AAAGCAGCTGCAAATACCAATG | AAATGTGTCTTCCAGTCGGTAG |
| Ppara | ACGATGCTGTCCTCCTTGATGAAC | GATGTCACAGAACGGCTTCCTCAG |
| Pgc1a | GGATATACTTTACGCAGGTCGA | CGTCTGAGTTGGTATCTAGGTC |
| Cpt1 | CTACATCACCCCAACCCATATT | GATCCCAGAAGACGAATAGGTT |
| Cpt2 | TGTCTTTGATGTCCTCGATCAA | TCGGTTCTCACTGGTCAAATAA |
| Acox1 | CCAATGCTGGTATCGAAGAATG | CGACTGAACCTGGTCATAGATT |
| Acox2 | CAATGACTTCCATCAAGTGGTG | GTCTATGTTTTCGAAGCCCATC |
| Gapdh | AATGGTGAAGGTCGGTGT | GTGGAGTCATACTGGAACATGTAG |
